# Modelling the physical properties change of canned glutinous rice porridge during cooking†

**DOI:** 10.1039/c8ra07790h

**Published:** 2019-02-13

**Authors:** Lei Wang, Mengting Wang, Ruiling Lv, Mingming Guo, Xingqian Ye, Tian Ding, Donghong Liu

**Affiliations:** College of Biosystems Engineering and Food Science, Zhejiang University Hangzhou 310058 China dhliu@zju.edu.cn; Fuli Institute of Food Science, Zhejiang University Hangzhou 310058 China; Zhejiang Key Laboratory for Agro-Food Processing, National Engineering Laboratory of Intelligent Food Technology and Equipment Hangzhou 310058 China

## Abstract

In this study, we modeled the water absorption, softening and shear viscosity change kinetics of canned rice porridge during cooking as well as estimated the thermodynamic properties involved in hydration. Moreover, the internal microstructure of rice kernels was observed under different hydrothermal conditions. During cooking, the water absorption and shear viscosity alteration rate increased with temperature, whereas the softening rate decreased. However, the temperature did not significantly affect the equilibrium value of the physical properties. The variation tendencies of the moisture content and hardness of the kernels could be expressed satisfactorily by the exponential and the generalized exponential models. The porridge shear viscosity variations fitted the sigmoidal and its generalized models. Thermodynamic parameters (enthalpy, entropy and Gibbs free energy) revealed that the hydration process was non-spontaneous and exothermic. Furthermore, scanning electron microscopy images and the results of the X-ray diffraction analysis showed the microstructure of the kernels during cooking, and the kernels formed a homogeneous mesh structure at earlier times during the initial stage at higher temperatures. These findings would provide valuable information for the optimization of canned rice porridge production.

## Abstract 2

The changes in the physical properties of rice porridge during cooking in a sealed can were investigated and modeled systematically.

## Graphical abstract

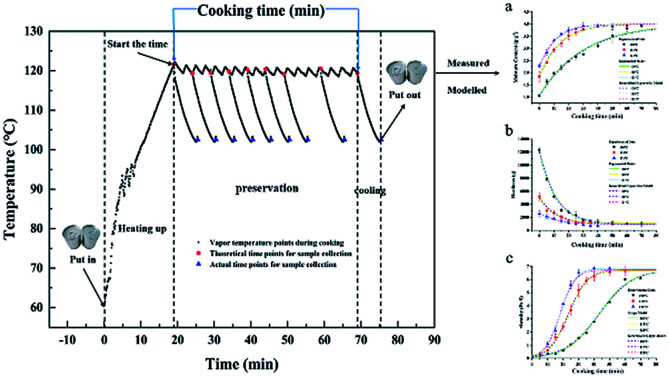

## Introduction

Rice porridge is a traditional food in Asia. It is usually prepared by adding water to the kernels (the ratio of water to rice, about 7 : 1 (v/w)) followed by heating for several hours until it transformed into a semi-liquid food;^1^ however, the cooked rice porridge can be stored for only several days at ambient temperature. In recent years, ready-to-eat (RTE) rice porridge has attracted significant attention in many countries because of its excellent storage stability.^2^ Particularly, canned rice porridge is more attractive, which not only meets the requirement of commercial sterilization, but could also be consumed right out of the can.

Nowadays, a number of people prefer cooked rice products, *i.e.* rice porridge and rice having good eating quality, which is strongly associated with its physical properties.^3,4^ Cooking of canned rice porridge simply needs excessive water and heat, and this process develops the physical properties, such as moisture content and hardness, by gelatinization of the starchy kernel fractions and leaching of the soluble starch components,^5^ which are important for rice porridge. To date, a large amount of research has been conducted to investigate and model the change in the moisture content,^6^ volume^7^ and hardness^5^ of the rice kernels during soaking or rice cooking; however, there is insufficiently disclosed information about the cooking process of canned rice porridge, which is similar but more complicated. Therefore, it is necessary to study the property changes of canned rice porridge during the cooking process.

If the main physical factors that influence the eating quality of the rice porridge are known, the cooking process can be optimized to achieve the best possible quality of the porridge product for the consumer. In this way, kinetic modelling can provide a deeper understanding of the changes that occur during hydrothermal processing and guarantee the canned rice porridge industry control and optimization of the process in an economical and convenient way.^8–11^

It's also worth studying the thermodynamic properties involved in the cooking process and microstructure changes of the kernels under different temperatures.^8^ According to Mir *et al.*,^12,13^ it's possible to obtain information on specifying morphological changes of the rice kernel and their influence on the final quality by physical structure analysis. And the study of the thermodynamic properties made it possible to understand the different forms of energies and phenomena involved in this process.^6^

Therefore, in this context, the change tendencies of the moisture content and the hardness of the rice kernels, and shear viscosity of rice porridge during the cooking process at different temperature are investigated. In addition, the exponential models or sigmoidal models are fitted to experimental data. The thermodynamic properties of the processes are also determined, as well as the microstructure changes of the starch granules in the rice kernels processed in different hydrothermal conditions.

## Materials and methods

### Materials

Polished glutinous rice kernels are obtained from Hangzhou Wahaha Group Co. Ltd. (Hangzhou, China).

### Porridge cooking

**Fig. 1:**
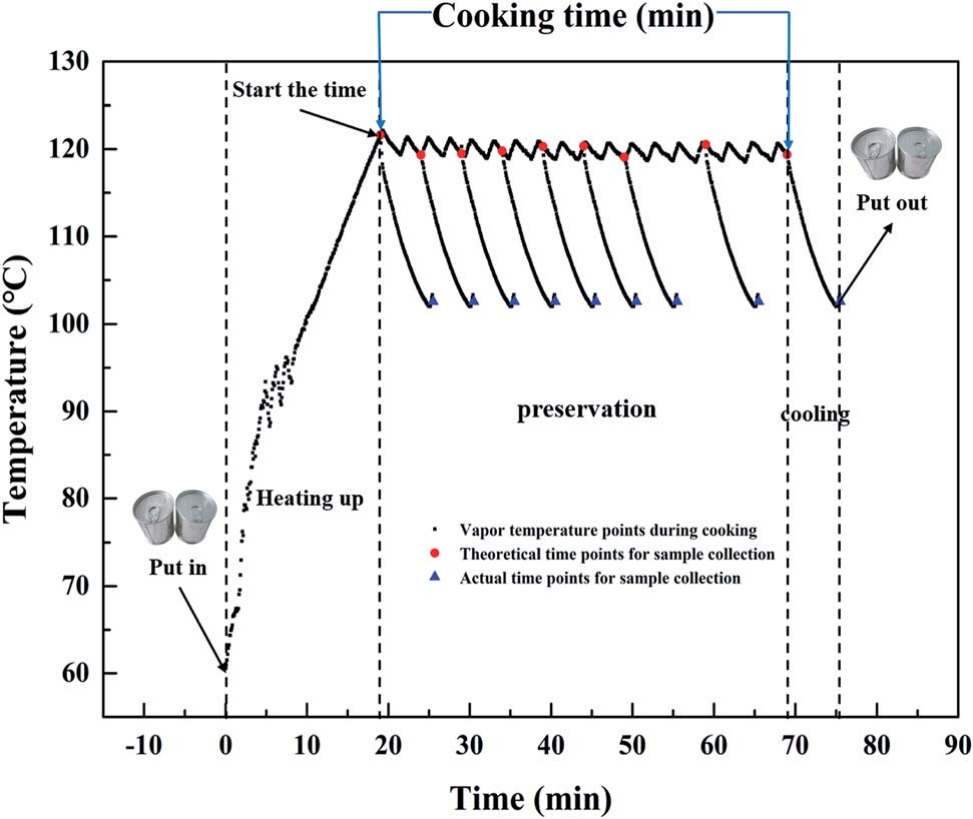
Temperature history and time points for sample collection (theoretical and actual) during cooking at 121 °C.

Canned rice porridge is prepared following the method of Kwang, *et al.*^14^ with some modifications. Polished glutinous rice (15 g) is washed and rinsed three times with tap water. Water is added to the rice with a ratio of 8 : 1 (v/w) in a commercial tin can (*ΦA* = 73 mm, *H* = 59.8 mm) (ORG Packaging Co. Ltd., Beijing, China), it is then sealed by hand seamer (YJ-C200, Zhangjiagang Yijie Automation Equipment Co., Ltd., Suzhou, China). The sealed can is put into an autoclave (Beijing Fanwen Trade Co. Ltd., Beijing, China) where the temperature is pre-heated to 60 °C. Then the autoclave is further heated up to 100 °C, 115 °C and 121 °C, maintained for 0–70, 0–60 and 0–50 min, respectively, and cooled down quickly by manual exhaust. The temperature changes of the vapor during cooking are monitored using MPIII Temperature Data Loggers (M4T12396, Mesa Laboratories, Inc., Lakewood, USA). Take the cooking process at 121 °C as an example, vapor temperature changes, and theoretical and actual time points for sample collection during cooking are shown in Fig. 1.

Specifically, only the preservation stage is investigated in this study, because the objective of this work is to model the continual physical properties changes in the rice porridge during cooking. Therefore, the cooking time mentioned in our study referred to the retention time shown on the display screen of the autoclave, which started the time when autoclave reached the setting temperature. In order to minimize the impact of the heating up and cooling stage on the changes of the physical properties during porridge cooking, the sealed can is put into an autoclave after temperature is pre-heated to 60 °C, and as soon as the vapor temperature dropped to 100 °C, the can is transferred to cold water for further cooling until it reached room temperature. The collection interval is 5 min when the cooking time is in the first 30 min, and thereafter, samples are collected every 10 min up to the equilibration of the hydration.

The cooking water of samples is withdrawn using a 20-mesh strainer and used for the analysis of the viscosity. The wet glutinous kernels are drained for weight analysis.^15^ Then some kernels are used for hardness analysis, and the rest of the kernels are freeze-dried, ground into powder, and pass through a 100-mesh sieve for analysis of the microstructure and the crystallinity of starch.

### Physical properties

#### Moisture content

In order to calculate the moisture content (g g^−1^ on a dry weight basis) of the cooked glutinous kernels at each time step, the water on the surface of the kernels is removed, then weighted at the corresponding time.^16^ The moisture content of the raw glutinous rice kernels is calculated using the standard AOAC method.^17^

#### Texture analysis

The cooked kernels are spread on a layer for texture measurement according to the instructions supplied with the texture analyser (TA-XT2i; Stable Micro Systems, Ltd., Surrey, UK), and the data obtained under the following conditions: operating mode: TPA determination; type of operation: compression; probe type: P50 probe; distance: 1.5 mm; number of cycles: 2 times; pre-test speed: 2 mm s^−1^; test speed: 0.5 mm s^−1^; post-speed: 5 mm s^−1^; the sensible stress is 5 g.

#### Rheological measurement

The relationship between shear stress and shear rate (0.01–100 s^−1^) of the cooking water of the glutinous rice are determined using a plate (C60/10° Ti L) and plate geometry rheometer (HAAKE RheoStress 6000; Thermo Fisher Scientific Co., Ltd., Shanghai, China) at 25 °C with 0.52 mm sample gap.

### Mathematical modelling

#### Water absorption

To simulate the water absorption during the cooking process of the canned rice porridge, the exponential model (eqn (1)) used by previous researchers for modelling the water uptake of the rice kernels during absorption process is investigated.^11,18,19^1
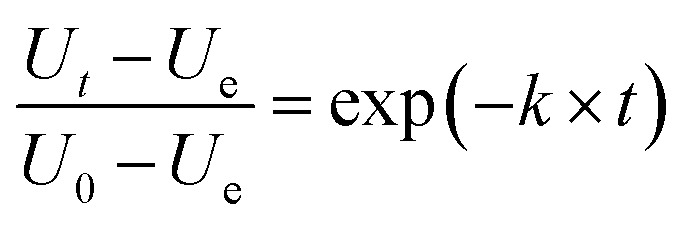
where *U*_*t*_ is the instantaneous moisture content; *U*_0_, the moisture content when the heating preservation time is 0 min; *U*_g_, the moisture content after prolonged cooking; *t*, the cooking duration, min; and *k* is the hydration constant.

The effects of temperature on the water absorption are expressed by an Arrhenius-type equation, eqn (2) as followed:2
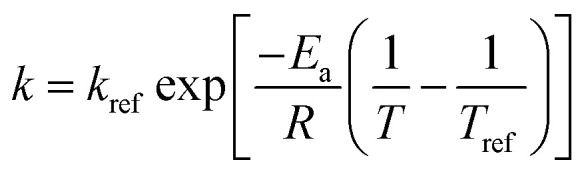
where *k*_ref_ is the hydration reference constant at a reference temperature; *E*_a_ is the activation energy for the hydration process (kJ mol^−1^); *R* is the universal gas constant (8.314 J mol^−1^ K^−1^); *T* and *T*_ref_ are hydration and reference temperatures (K), respectively. The *T*_ref_ employed here is 385.15 K (the average of the temperatures used for cooking).

It should be mentioned that *U*_0_ is not the same at different cooking conditions, which is mainly related to the temperature and time. All air in the autoclave would be excluded when the temperature is at 100 °C, then the distribution of the internal temperature is uniform. After that, the heating rate will be a constant when the temperature is increased from 100 °C to 121 °C, *i.e.* the temperature can be considered as a linear function with increasing time (Fig. 1). As a consequence, it is suitable to associate *U*_0_ as a one-variable function of temperature (eqn (3), Fig. S1a†).3
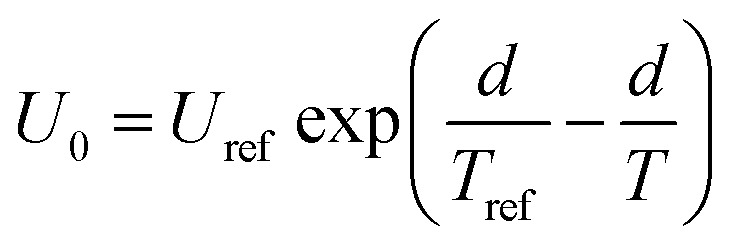
where *U*_ref_ is the moisture content when heat preservation is 0 min at the reference temperature; *d*, the regression coefficients.

Substituting eqn (2) and (3) into (1), the generalization of the exponential model (eqn (3)) is obtained, and is capable of predicting the amount of water absorbed for different thermal process conditions. The parameter 
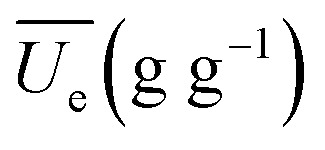
 is the arithmetic mean of the values of the *U*_e_ at different temperatures. The generalized equation is solved using nonlinear regression.4
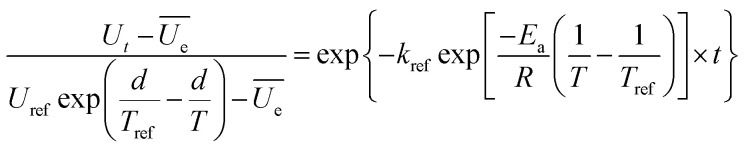


#### Softening

The glutinous rice normalized hardness (instantaneous hardness of rice kernel during cooking/the hardness of raw rice)^20^ values are fitted using the exponential model (eqn (5)) proposed by Miano, *et al.*^21^5
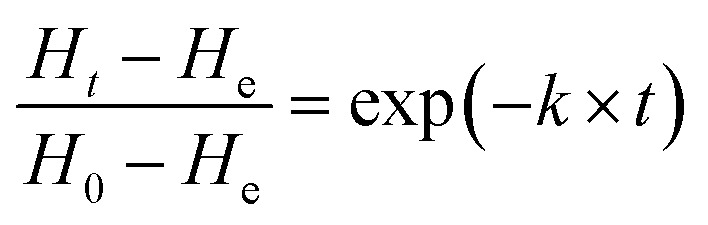
where *H*_*t*_ is the instantaneous normalized hardness; *H*_0_, the normalized hardness when the heating preservation time is 0 min; He, the normalized hardness after prolonged cooking; *t*, the cooking duration, min, and *k* is the softening constant.

In this study, the influence of the temperature on *H*_0_ is expressed by eqn (6) (Fig. S1b†).6
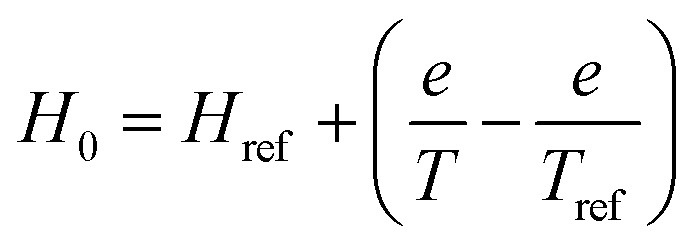
where *H*_ref_ is the hardness when autoclave reached reference temperature, and *e* is the regression coefficients.^3^

In order to predict the hardness alternation at different temperatures during the process, the generalization of the exponential model (eqn (7)) is chosen.7
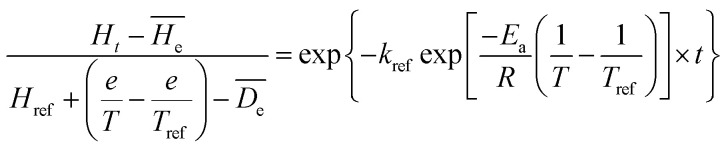
where *k*_ref_ is the softening reference constant at a reference temperature; *E*_a_ is the activation energy for the softening process (kJ mol^−1^); *R* is the universal gas constant (8.314 J mol^−1^ K^−1^); *H*_m_, the initial hardness; *H*_ref_, the hardness when heat preservation is 0 min at reference temperature, *T* and *T*_ref_ are hydration and reference temperatures (K), respectively.

#### Rheological changes

Many processes exhibit a progression from a small start which accelerates and approaches a climax over time. When a specific mathematical model is lacking, a sigmoid function is often used.^22^ The sigmoidal function has already been used to model the storage modulus changes of chicken breast meat,^23^ white kidney beans dehydration kinetics^24^ and hydration kinetics^21^ during thermal treatment. In this section, the sigmoidal equation (eqn (8)) is used to fit the trends of shear viscosity changes of samples during cooking at 100 °C, 115 °C and 121 °C.8
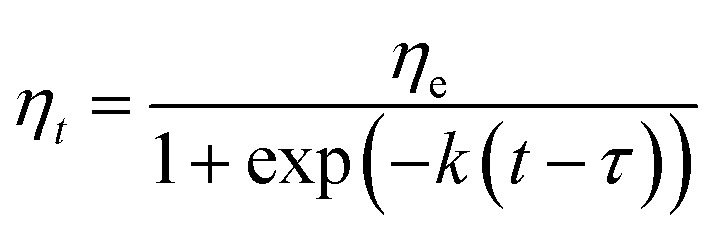
where *η*_*t*_ (Pa s) is the instantaneous shear viscosity; *η*_e_ (Pa s), the equilibration shear viscosity; *t* (min), the cooking duration; *k*, the constant rate of cooking, and the parameters *τ* (min) is defined as the time need to obtain half equilibration viscosity of the cooking water.

An Arrhenius-type equation is also used to describe the influence of temperature shear viscosity kinetics constant *c*.9
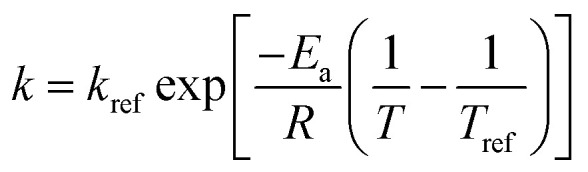
where *c*_ref_ is the softening reference constant at a reference temperature; *E*_a_ is the activation energy for the hydration process (kJ mol^−1^); *R* is the universal gas constant (8.314 J mol^−1^ K^−1^); *T* and *T*_ref_ are hydration and reference temperatures (K), respectively.

Substituting eqn (9) into (8), the generalization of the model (eqn (10)) is obtained, which is able to predict the shear viscosity at any temperature and time during the process. The parameter *ā* and *b̄* are the arithmetic mean of the values of *a* and *b* at different temperatures.10
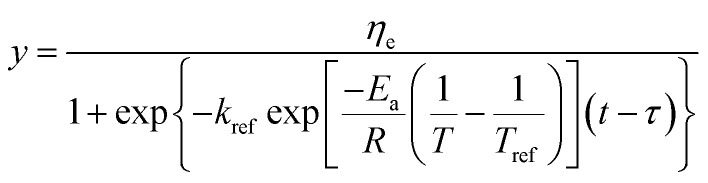


### Thermodynamic properties

The linearization of eqn (2) yields eqn (11), from which the activation energy value can be calculated.11
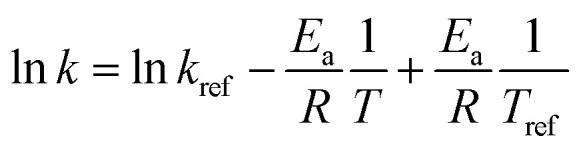


Other thermodynamic parameters of the hydration process, such as enthalpy Δ*H* (kJ mol^−1^), entropy Δ*S* (kJ mol^−1^ K^−1^) and Gibbs free energy Δ*G* (kJ mol^−1^) are determined from the model eqn (12), (13) and (14), respectively.

12 Δ*H* = −(*E*_a_ + *RT*)

13
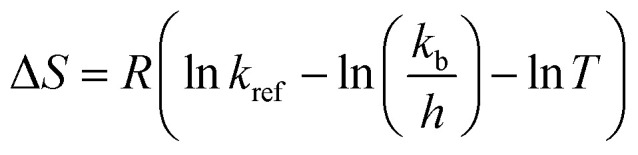

14 Δ*G* = Δ*H* − *T*Δ*S*

In these equations, *R* is the universal gas constant (8.314 J mol^−1^ K^−1^), *k*_b_ is Boltzmann's constant (1.38 × 10^−23^ J K^−1^), *h* is Planck's constant (6.626 × 10^−34^ J s), *T* and *T*_ref_ is the temperature (K), *k* and *k*_ref_ is the hydration constant and the hydration constant at a reference temperature *T*_ref_, respectively.

### Morphology property observation

The cross-section of the cooked kernels during the period of cooking is observed by a scanning electron microscope (SEM). The freeze-drying kernels are mounted onto a stub with double sided sticky tape, sputter coated with gold in a sputter coater (SCD 050, Balzers, Liechtenstein), and then observed using a Tabletop Microscope (TM-1000, HITACHI, Japan).

### X-ray diffraction analysis

The kernels flour is exposed to an X-ray beam at 40 kV and 40 mA, and scanned from 4 to 40° 2*θ* with a step size of 0.02° using an X-ray powder diffractometer (XRD) (X′ Pert PRO, PANalytical B.V., Netherlands).

### Statistical analysis

The data are fitted by MATLAB Software Version R2015a (The Math Works, Inc., MA, USA). The figures are plotted by Origin Software Version 9.1 (Origin Lab Corp., MA, USA).

## Results and discussion

### Physical properties

It's worth noting that the time required for the autoclave to reach 100 °C, 115 °C and 121 °C is 8 min, 15.5 min and 18.5 min, respectively, so the moisture content, hardness and shear viscosity varies with different temperatures at starting time.

### Moisture content

**Fig. 2:**
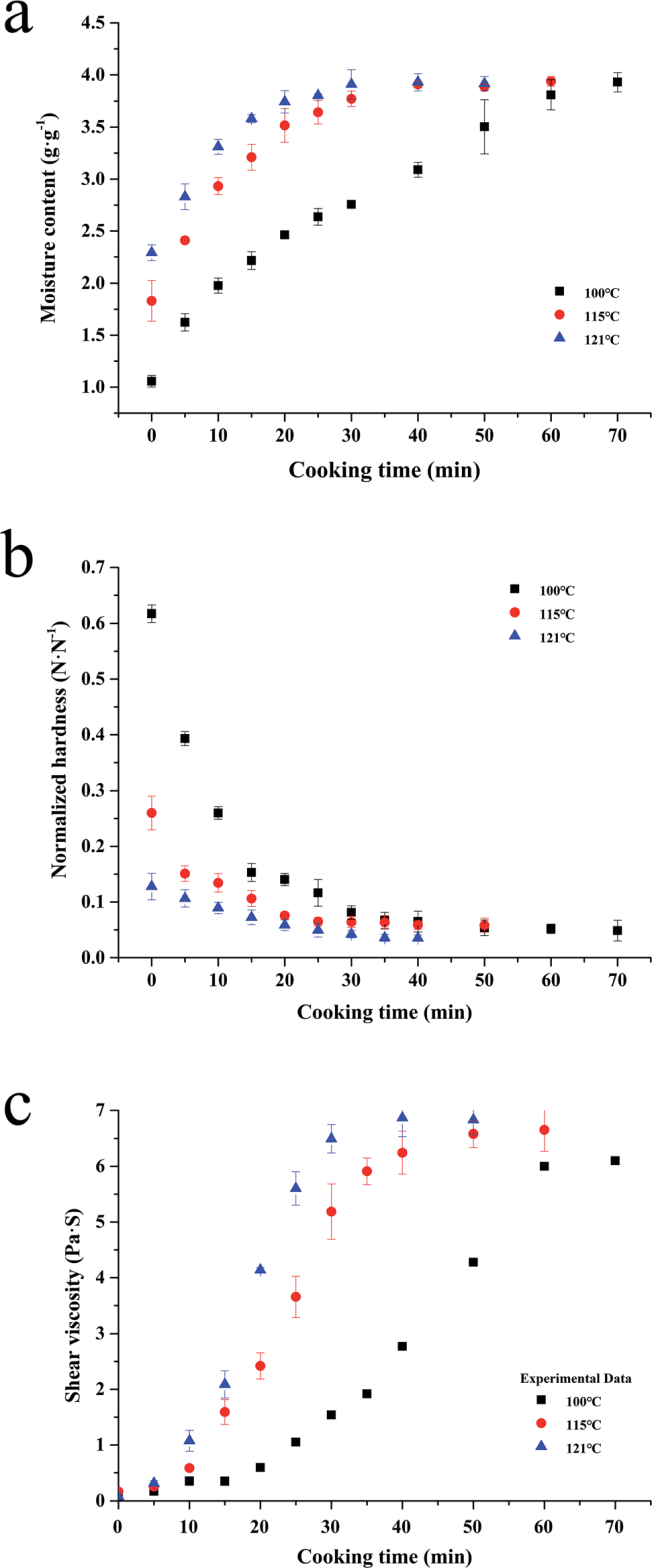
Water absorption (a), softening (b) and shear viscosity (c) kinetics of canned rice porridge under different thermal conditions (100–121 °C) during cooking.

Water absorption is usually regarded as a water diffusion process, caused by the moisture gradient between the system and the kernels. Moreover, this process is also affected by the system temperature and the varieties of rice.^10^ And as the rice solids are leached, the consistency of the fluid increased, also affecting the grain hydration. As shown in Fig. 2a, the influence of the system temperature on the process can be seen, which indicates that the water absorption rate increases with increased cooking temperature (Fig. 2a). This phenomenon might be partly due to more molecular bonds being broken at a higher temperature, which are able to form water–hydrogen bonds.^10^

Although the water absorption rate increased with the increment of temperature in the whole process, the alteration tendency of the moisture content at different sterilization temperatures is similar. During the initial period of cooking (0–10 min for 100 °C, 0–20 min for 115 °C and 0–50 min for 100 °C), the absorbed water increased sharply. As the process continued, the water absorption rate became slower compared to that at the beginning of the cooking process. The water absorption ceased when the rice kernels reached the maximum moisture content (3.99 g g^−1^), and the saturation moisture content is nearly a constant. Similar results have been reported for the temperature having no obvious effect on the equilibrium moisture content.^25,26^

### Hardness

Hardness is the most important texture parameter,^27^ which is relevant to the quality of the cooked rice kernels.^5^ The changes of the kernels normalized hardness at different temperatures are shown in Fig. 2b. An initial high rate of kernels softening is followed by slower softening in later stages, which is similar to the characteristic softening behaviour.^28^ With the cooking duration, the decrease of the normalized hardness became unnoticeable after reaching a minimum level (0.05). It could be caused by the increase of moisture content (Fig. 1a) and the pore size formed by starch gelatinization (Fig. 4) within the rice kernels during cooking.^21,28^ This softening tendency of the rice kernels is consistent with that of the rice kernels during cooking at 95 °C reported by Nawaz, *et al.*^5^ Remarkably, a much lower normalized hardness value (0.13 N N^−1^ at 121 °C, but 0.62 N N^−1^ at 100 °C) and slower softening rate occurred at higher temperatures when the cooking time is 0 min; this is caused by the longer rise time required for the autoclave to reach the target temperature.

### Shear viscosity

Shear viscosity of a liquid fraction of porridge is a very important rheological characteristic of rice porridge, and is of great significance in processing, quality control, sensory evaluation and consumer acceptability.^29^ In order to study shear viscosity changes during porridge cooking quantitatively, a series of shear viscosity data are collected by maintaining the shear rate at 0.1 s^−1^. As can be seen in Fig. 2c, the trend of the porridge shear viscosity changes exhibit a sigmoidal behaviour at different temperatures, and the rate of shear viscosity change at 100 °C is significantly lower than that at 115 °C and 121 °C.

It's well known that the shear viscosity of a porridge depends on the amount of amylopectin leaching from the starch granules into water,^30^ and the amount of water absorbed by the rice kernels. Hence, trends of the kinetic curves coinciding with a sigmoidal behaviour may be ascribed to two comprehensive factors. One is that the rice kernels contain fine cracks throughout the kernels, and these cracks become wider when the kernels are cooked.^31^ The other is that the starch granules will gradually gelatinize and adhere together during the process of cooking.^15^

In the present study, the starch granules in rice kernels gelatinized and formed a mesh structure during glutinous porridge cooking, so that the cracks became more defined, and consequently, the water could easily penetrate into the kernels and the amylopectin in the glutinous rice easily leach out into the water. In the beginning, the starch granules absorbed water and swelled, so there are no obvious changes in the shear viscosity of the porridge until the breakdown of the starch granules. While the amount of remaining amylopectin in the rice kernels declined, the shear viscosity of the porridge would reach a maximum at the end, because of the gelatinized starch adhering together.

### Mathematical modelling of physical properties

Moisture content, the hardness of the rice kernels and shear viscosity of the porridge during the cooking process are determined, and the experimental data are fitted to the models and generalized models respectively (Fig. 2). The performances of the models are determined according to their coefficient of determination (*R*^2^), the root mean square error (RMSE, %) and the mean relative deviation modulus (*P*). Furthermore, these generalized model equations can be used in the modeling of thermal conditions other than those investigated in the present study, leading to time and cost minimization.

### Water absorption and softening

**Fig. 3:**
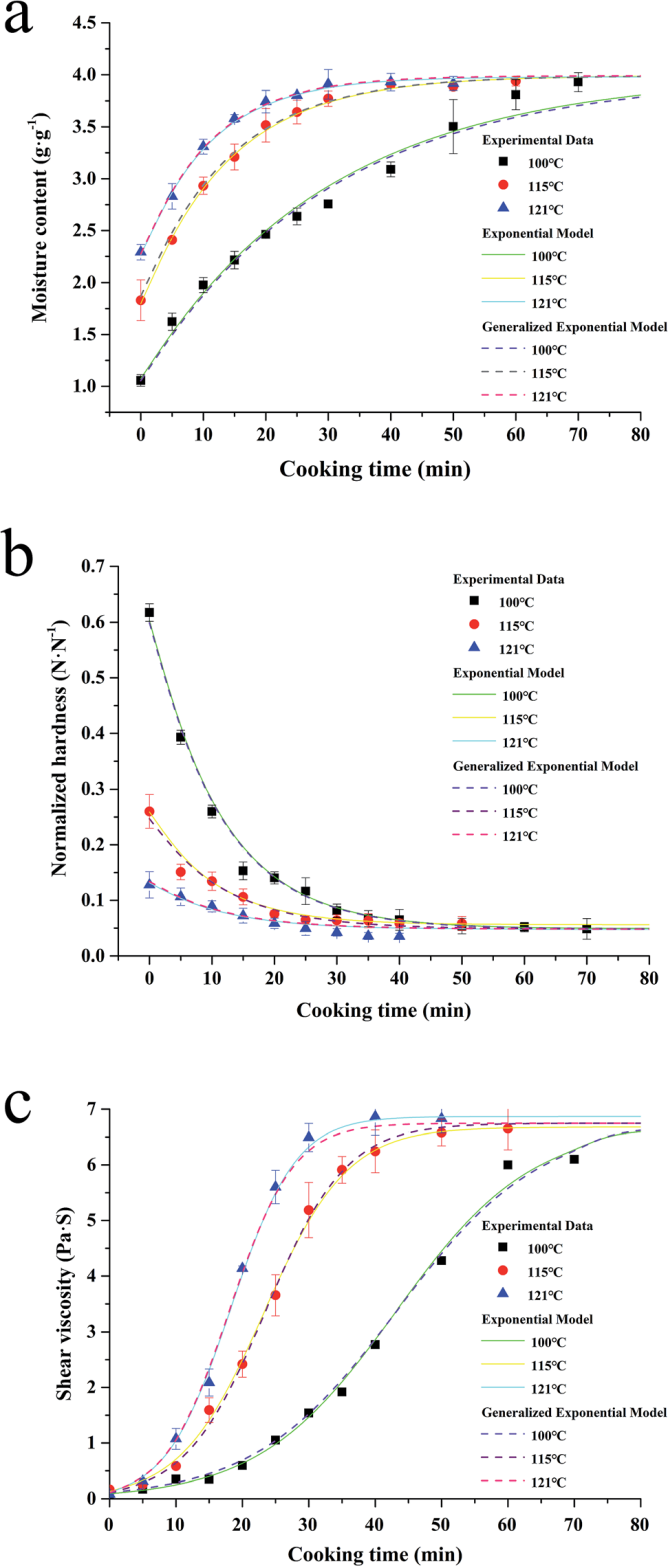
Fitting of the exponential model together with its generalization (a & b), and sigmoidal model together with its generalization (c) to moisture content, normalized hardness and shear viscosity of canned rice porridge respectively during the cooking process under different sterilization temperatures (100–121 °C).

The exponential model is one of the most common empirical models to simulate the water uptake process of agricultural products.^24,32^ The models (eqn (1) and (4)) are reasonably fitted to the experimental data from the process of water uptake during the sterilization (Fig. 3a), with the *R*^2^ ranging from 0.979 to 0.997, RMSE ranging from 0.041 to 0.137 g g^−1^, and *P* below 3.65%, as shown in Table 1. Similar RMSE values have been reported by Kashaninejad, *et al.*,^18^ ranging from 0.073 to 0.114 g g^−1^, when the exponential model is applied to model the data from the parboiling of white rice in the temperature range of 25–70 °C.

**Table 1:** Statistical analysis of the fitting of the models to different moisture content data of glutinous rice kernel (100–121 °C) during porridge cooking

*T* (°C)	*R* ^2^		*P* (%)		RMSE (g g^−1^)	
	E^a^	EG^b^	E	EG	E	EG
100	0.981	0.979	3.650	3.546	0.137	0.130
115	0.997	0.995	1.051	1.367	0.042	0.050
121	0.996	0.995	0.871	0.887	0.043	0.041

a Exponential model.

b Generalized exponential model.

The hydration constant, coefficient *k*, which is related to the effective diffusivity during the hydration process, is sensitive to temperature variations (3.342 × 10^−2^ to 9.390 × 10^−2^ s^−1^). This finding is consistent with the results reported by (ref. 33) that the hydration constant of red lentil cultivar PUSA is between 0.877 and 1.495 h^−1^ at a soaking temperature range of 15 to 45 °C. Similarly, the hydration coefficient *k* is from 3.2 × 10^−2^ to 5.7 × 10^−2^ s^−1^ when milled whole kernels of 10 rice cultivars with 12% initial moisture levels are soaked at 25 °C.^19^ The value of this parameter varied between the rice and lentil due to the characteristics of the products and the process.

The model (eqn (5) and (7)) demonstrated the softening of rice kernels under different thermal conditions (Fig. 3b), presented an average *R*^2^ = 0.977, RMSE = 0.01 N N^−1^, and *P* = 6.994%, as shown in Table 2. According to Mcminn, *et al.*^34^ and Mpotokwane, *et al.*,^35^*P* value of less than 10% indicated a good fit for practical purposes. Therefore, the exponential model can be effectively used to describe the trends of the hardness of glutinous rice during the porridge process.

**Table 2:** Statistical analysis of the fitting of the models to different normalized hardness data of glutinous rice kernel (100–121 °C) during porridge cooking

*T* (°C)	*R* ^2^		*P* (%)		RMSE (N N^−1^)	
	E^a^	EG^b^	E	EG	E	EG
100	0.994	0.994	5.383	5.165	0.014	0.014
115	0.975	0.973	5.691	8.004	0.010	0.011
121	0.965	0.960	8.604	9.114	0.006	0.006

a Exponential model.

b Generalized exponential model.

### Shear viscosity variation

In this study, the sigmoidal model (eqn (8) and (10)) is applied to describe a similar change of the shear viscosity of the porridge during cooking (Fig. 3c), with *R*^2^ ranging from 0.995 to 0.999, RMSE ranging from 0.089 to 0.151 Pa s, and *P* values ranging from 7.495 to 9.553%, as shown in Table 3.

**Table 3:** Statistical analysis of the fitting of the models to different shear viscosity data (shear rate is 0.1 s^−1^) of glutinous rice kernel (100–121 °C) during porridge cooking

*T* (°C)	*R* ^2^		*P* (%)		RMSE (Pa s)	
	E^a^	EG^b^	E	EG	E	EG
100	0.996	0.995	8.305	9.553	0.151	0.158
115	0.997	0.997	8.431	7.495	0.125	0.148
121	0.999	0.998	8.386	8.342	0.089	0.121

a Sigmoidal model.

b Generalized model.

### Thermodynamic properties of the process

It is well-known that the increase of the reaction medium temperature usually increases the reaction rate. This is due to the excess heat in the system, which is responsible for the more random movement of the reactant particles, leading to an increase in the interactions. The rate of reaction depends on the interactions between the reacting molecules. As is shown in Table 4, the activation energy (61.91 kJ mol^−1^) obtained from the generalization of the exponential model is very close to that of rough rice (65.4 kJ mol^−1^) in the range of 85–120 °C,^36^ and that of white rice (59.6 kJ mol^−1^) hydrated from 63–85 °C,^37^ but much higher than that of rough rice (30 kJ mol^−1^) at 60–90 °C.^38^ The activation energy varied between the varieties due to the different processing conditions used, as well as to the different composition and structure of the kernels.

**Table 4:** Thermodynamic parameters of the hydration of barley

*T* (°C)	*E*_a_ (kJ mol^−1^)	Δ*H* (kJ mol^−1^)	Δ*S* (kJ mol K^−1^)	Δ*G* (kJ mol^−1^)
100	61.91	65.01	−0.270	35.7
115		65.14	−0.270	39.7
121		65.19	−0.271	41.4

The enthalpy change (Δ*H*) could be adopted to describe the heat released during water absorption by the kernel, and thus the binding energy or the intercellular force between water and the molecules in the kernel are calculated.^39^ Under our condition, the values of Δ*H* (−65.01 to −65.19 kJ mol^−1^) were all negative in Table 4, indicating the hydration process has a favourable exothermic change.^9,34^

The entropy (Δ*S*) values (−0.270 to −0.271 kJ mol K^−1^) are also obtained (Table 4), and an endothermic process is reflected by the negative values. In addition, the values in our study are nearly constant with increased temperature, indicating that this process occurred without significant disorder of the system. These findings are in agreement with the results of Balbinoti, *et al.*,^8^ who studied the hydration process of rice (*Oryza sativa*) and found that the value is −0.273 kJ mol K^−1^.

The positive values for the free energy change (Δ*G*) in our present study suggested that the cooking process is thermodynamically favourable and did not occur spontaneously. Meanwhile, the value for Δ*G* increased (35.7–41.4 kJ mol^−1^) with increments in the cooking temperature due to the enthalpy and entropy changes (Table 4). According to the report of Mcminn, *et al.*,^34^ the Δ*G* values could illustrate the water affinity of the food. That is the reason that rice tended to absorb water more easily at higher treatment temperatures, which was also mentioned in the section of moisture content.

### Microstructure changes during cooking

**Fig. 4:**
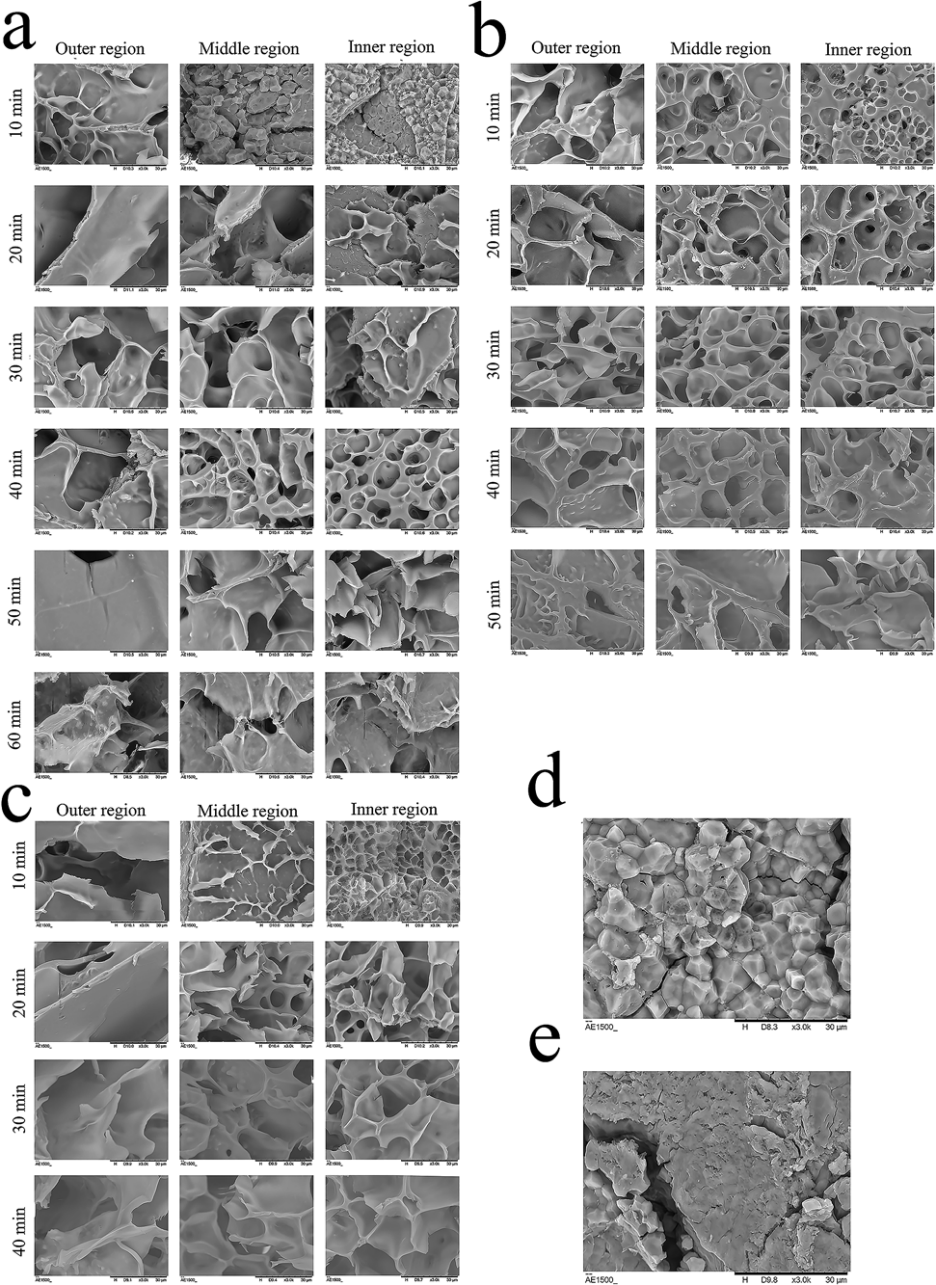
Scanning electron microscopy images of different regions of kernels during the cooking process of canned glutinous porridge at 100 °C (a), 115 °C (b) and 121 °C (c) and raw glutinous rice (d and e).

Starch is the major component in glutinous rice, and the physical properties of glutinous porridge are strongly affected by the gelatinization of starch.^15^ In order to investigate the structural change of starch granules corresponding to that of the physical properties (Fig. 4), a cross section of the raw and cooked glutinous rice kernels is observed using SEM at 10 min interval during the cooking process of porridge. Unlike the changes in the starch granules during the gelatinization, which are divided into three periods,^15^ the changes of starch granules during porridge cooking are divided into four periods: granule swelling, morphological change, granule adhesion, and the increase of pore size (Fig. 4).

Glutinous rice starch granules are homogeneous in shape (polyhedral structure),^15^ but the starch granules in the middle and inner region of the kernels are packed more tightly than in the outer region (Fig. 4d and e). As shown in Fig. 3a–c, the microstructure of starch granules in the rice kernels changed in varying degrees during cooking at 100 °C, 115 °C and 121 °C, respectively. When cooking at 100 °C for 10 min, the starch granules in the outer region of the kernels adhered together and formed a three-dimensional mesh structure; the starch granules in the middle region swelled but maintained the polygonal shape, but the starch granules in the inner region of the kernels kept their shape intact similar to uncooked kernels. After 20 min of cooking, the pore size of the paste starch in the outer region got larger, and the starch granules in the middle region of the kernels changed shape and adhered together, but most of the starch granules in the inner granule region of the kernels are still tightly packed. After 30 min of cooking, the starch granules in the inner region of the kernels formed a mesh structure partly, whereas other areas exhibited a mesh structure with a large pore size. After 40 min of cooking, all the starch granules in the kernels formed a mesh structure but the pore size in the inner region is smaller than in the middle and outer regions. Up till 60 min of cooking, all the starch granules in the kernels formed a homogeneous mesh structure. When the cooking temperature is 115 °C, all the starch granules in the kernels formed a homogeneous mesh structure at 40 min (Fig. 3b). At a temperature of 121 °C, all the starch in the kernels formed a homogeneous mesh at 30 min (Fig. 4c). The quicker the kernels formed a homogeneous mesh structure, the quicker the kernel absorbs water. This phenomenon can be also be proven by the Δ*G* values. Pan, *et al.*^15^ reported similar behaviour on rice cooking.

Therefore, the results indicate that one suitable processing condition for the glutinous rice porridge is cooking at 121 °C for 30 min, where the kernels reached a moisture content of 3.91 g g^−1^ (dry basis) and had formed a homogeneous mesh structure. Simultaneously, the expected characteristics (hardness and shear viscosity) are very close to the equilibrium values.

### X-ray diffraction analysis

**Fig. 5:**
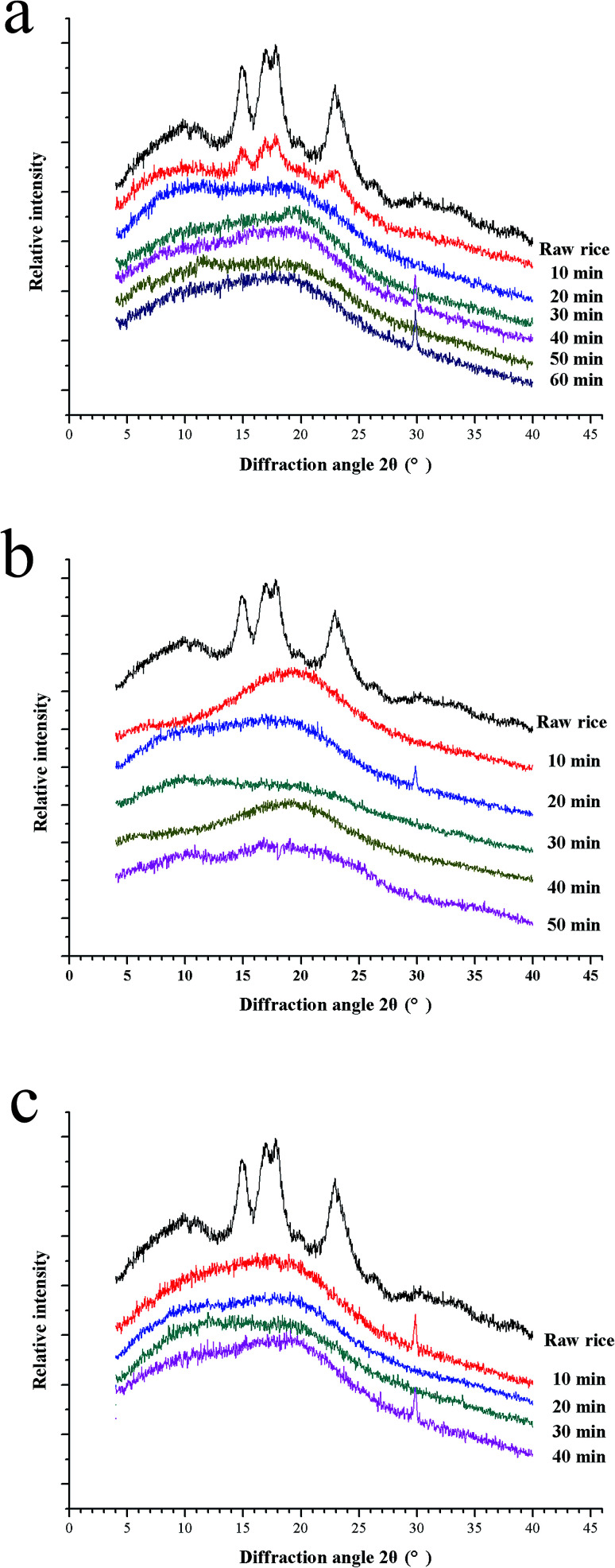
X-ray diffraction patterns 100 °C (a), 115 °C (b) and 121 °C (c). Profile of each measurement is shifted apart from each other for the purpose of clarity.

The crystalline structure of starch in glutinous kernels is investigated using XRD during the cooking process at different temperature (Fig. 5a–c). The starch in uncooked glutinous kernels exhibited a typical A-type XRD pattern with strong diffraction peaks at 2*θ* = 15°, 17°, 18° and 23°, which is consistent with that of normal cereal starches.^40^ It is well known that the crystalline degree of the starch decreases during cooking. Compared to raw glutinous rice, the characteristic peaks of cooked glutinous rice kernels are obviously weakened, which implied their crystalline structure changed dramatically during the porridge cooking process. It can be seen that the crystalline structure of starch in glutinous rice has been completely destroyed at 20 min, 10 min and 10 min when the temperature is 100 °C, 115 °C and 121 °C, respectively (Fig. 5). These results are consistent with that of the SEM, which showed that the starch granules completely gelatinize at 20 min, 10 min and 10 min at the corresponding temperature (Fig. 4). In addition, it can be also proven that the physical changes of the rice porridge occurred mainly after the entirely gelatinization.

## Conclusion

The physical properties are important for the eating quality of canned rice porridge, however, the related research is missing in literature. Therefore, it is necessary to get information on the evolution of the cooking of canned porridge so as to precisely quantify the cooking procedure. This work presented detailed changes in the physical properties of canned rice porridge during cooking at 100 °C, 115 °C and 121 °C, and the microstructure of the starch granules in the kernels. Since gelatinization occurs entirely during the initial period of cooking, the internal structure and physical properties including the moisture content and the hardness of the kernels, as well as the shear viscosity of the rice porridge are also dramatically influenced by the process temperature and time. But an increase in the temperature has no noticeable effect on the equilibrium value of the moisture content (3.99 g g^−1^), hardness (0.05 N N^−1^) and shear viscosity (6.747 Pa s). The tendencies for change of the moisture content and hardness of the kernels can be modelled well by exponential models with relative error lower than 3.650% and 8.604%, respectively. And sigmoidal models with a relative error lower than 8.386% is found to describe the trends of the shear viscosity of glutinous rice. The enthalpy (−65.01 to −65.19 kJ mol^−1^), entropy (−0.270 to −0.271 kJ mol^−1^ K^−1^) and free energy (35.7–41.4 kJ mol^−1^) obtained in this study demonstrated that the hydration of the canned porridge during the cooking process didn't occur spontaneously with increasing temperature. These results could contribute to the prediction and optimization of the eating quality of canned rice porridge.

## Conflicts of interest

The authors declare that no conflicts of interest exist.

## Supplementary Material

RA-009-C8RA07790H-s001
