# Supplementary material for: Modelling the physical properties change of canned glutinous rice porridge during cooking
Source: RSC Adv. 2019 Feb 13;9(10):5521–9. doi: 10.1039/c8ra07790h (PMC9060801; doi:10.1039/c8ra07790h)

Electronic Supplementary Material (ESI) for RSC Advances.
This journal is © The Royal Society of Chemistry 2018

The relationships between sterilization temperature and D_0 (Moisture content (Fig. S1); Normalized hardness (Fig. S2)) were acquired by linear regression.

Fig. S1

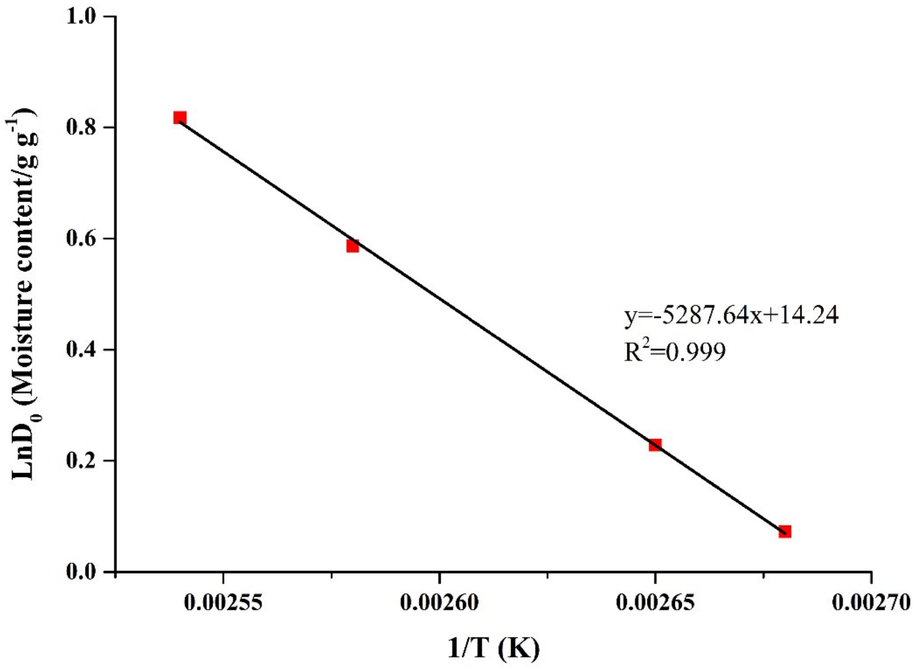

Fig. S2

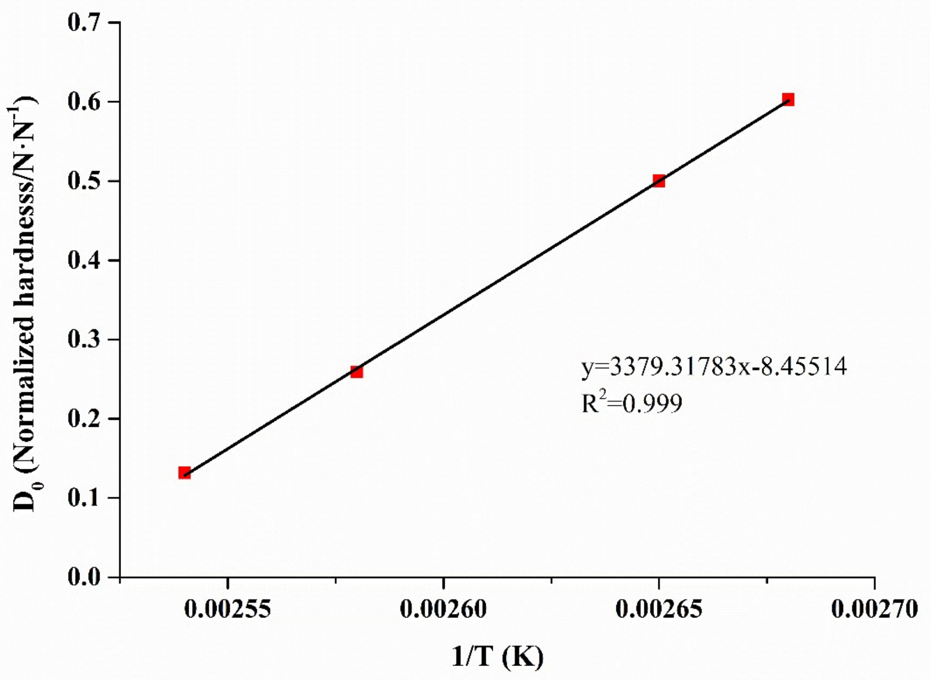

Supplement: RA-009-C8RA07790H-s001 [file RA-009-C8RA07790H-s001.pdf]
